# Diagnostic and therapeutic approaches for nonmetastatic breast cancer in Canada, and their associated costs

**DOI:** 10.1038/sj.bjc.6690228

**Published:** 1999-03

**Authors:** B P Will, C Le Petit, J-M Berthelot, E M Tomiak, S Verma, W K Evans

**Affiliations:** The Health Analysis and Modelling Group, Statistics Canada, 24-Q, R. H. Coats Building, Ottawa, Ontario, K1A OT6, Canada; and; The Ottawa Regional Cancer Centre, Cancer Care Ontario and the University of Ottawa, 501 Smyth Road, Ottawa, Ontario, K1H 8L6, Canada

**Keywords:** breast cancer, cost, microsimulation model

## Abstract

In an era of fiscal restraint, it is important to evaluate the resources required to diagnose and treat serious illnesses. As breast cancer is the major malignancy affecting Canadian women, Statistics Canada has analysed the resources required to manage this disease in Canada, and the associated costs. Here we report the cost of initial diagnosis and treatment of nonmetastatic breast cancer, including adjuvant therapies. Treatment algorithms for Stages I, II, and III of the disease were derived by age group (< 50 or ≥ 50 years old), principally from Canadian cancer registry data, supplemented, where necessary, by the results of surveys of Canadian oncologists. Data were obtained on breast cancer incidence by age, diagnostic work-up, stage at diagnosis, initial treatment, follow-up practice, duration of hospitalization and direct care costs. The direct health care costs associated with ‘standard’ diagnostic and therapeutic approaches were calculated for a cohort of 17 700 Canadian women diagnosed in 1995. Early stage (Stages I and II) breast cancer represented 87% of all incident cases, with 77% of cases occurring in women ≥ 50 years. Variations were noted in the rate of partial vs total mastectomy, according to stage and age group. Direct costs for diagnosis and initial treatment ranged from $8014 for Stage II women ≥ 50 years old, to $10 897 for Stage III women < 50 years old. Except for Stage III women < 50 years old, the largest expenditure was for hospitalization for surgery, followed by radiotherapy costs. Chemotherapy was the largest cost component for Stage III women < 50 years old. This report describes the cost of diagnosis and initial treatment of nonmetastatic breast cancer in Canada, assuming current practice patterns. A second report will describe the lifetime costs of treating all stages of breast cancer. These data will then be incorporated into Statistics Canada's Population Health Model (POHEM) to perform cost-effectiveness studies of new therapeutic interventions for breast cancer, such as the cost-effectiveness of day surgery, or of radiotherapy to all breast cancer patients undergoing breast surgery. © 1999 Cancer Research Campaign


*British Journal of Cancer* (1999) **79**(9/10), 1428–1436
© 1999 Cancer Research Campaign
Article no. bjoc.1998.0228

### Diagnostic and therapeutic approaches for nonmetastatic breast cancer in Canada, and their associated costs

**BP Will[1], C Le Petit[1], J-M Berthelot[1], EM Tomiak[2], S Verma[2] and WK Evans[2]**

[1]The Health Analysis and Modelling Group, Statistics Canada, 24-Q, R. H. Coats Building, Ottawa, Ontario, K1A OT6, Canada; and [2]The Ottawa Regional Cancer Centre, Cancer Care Ontario and the University of Ottawa, 501 Smyth Road, Ottawa, Ontario, K1H 8L6, Canada

**Summary** In an era of fiscal restraint, it is important to evaluate the resources required to diagnose and treat serious illnesses. As breast cancer is the major malignancy affecting Canadian women, Statistics Canada has analysed the resources required to manage this disease in Canada, and the associated costs. Here we report the cost of initial diagnosis and treatment of nonmetastatic breast cancer, including adjuvant therapies. Treatment algorithms for Stages I, II, and III of the disease were derived by age group (< 50 or ≥ 50 years old), principally from Canadian cancer registry data, supplemented, where necessary, by the results of surveys of Canadian oncologists. Data were obtained on breast cancer incidence by age, diagnostic work-up, stage at diagnosis, initial treatment, follow-up practice, duration of hospitalization and direct care costs. The direct health care costs associated with ‘standard’ diagnostic and therapeutic approaches were calculated for a cohort of 17 700 Canadian women diagnosed in 1995. Early stage (Stages I and II) breast cancer represented 87% of all incident cases, with 77% of cases occurring in women ≥ 50 years. Variations were noted in the rate of partial vs total mastectomy, according to stage and age group. Direct costs for diagnosis and initial treatment ranged from $8014 for Stage II women ≥ 50 years old, to $10 897 for Stage III women < 50 years old. Except for Stage III women < 50 years old, the largest expenditure was for hospitalization for surgery, followed by radiotherapy costs. Chemotherapy was the largest cost component for Stage III women < 50 years old. This report describes the cost of diagnosis and initial treatment of nonmetastatic breast cancer in Canada, assuming current practice patterns. A second report will describe the lifetime costs of treating all stages of breast cancer. These data will then be incorporated into Statistics Canada’s Population Health Model (POHEM) to perform cost-effectiveness studies of new therapeutic interventions for breast cancer, such as the cost-effectiveness of day surgery, or of radiotherapy to all breast cancer patients undergoing breast surgery.

**Keywords:** breast cancer; cost; microsimulation model

In 1995, it is estimated that 17 700 women of a total population of 14.9 million Canadian women were diagnosed with breast cancer, a 15% increase over the 15 455 cases diagnosed in 1993 (Statistics Canada, 1995; National Cancer Institute of Canada, 1998). This is more than twice the estimated number of cases of female lung (7300) or colorectal (7500) cancer, the next two most common cancers for Canadian women (National Cancer Institute of Canada, 1995). As breast cancer is the major malignancy affecting women, and therefore, a serious health care problem in Canada, it was thought useful to determine the resources used in breast cancer management (Will et al, 1993), based on the incidence of the disease, its management by stage over time, and the costs of the components of care. As breast cancer is a chronic, systemic disease (Tabar et al, 1992), a full understanding of resource consumption must consider the therapeutic options at first diagnosis, as well as the management of recurrent or metastatic disease, and of terminal care.

This paper summarizes the principal costs associated with the initial treatment of the nonmetastatic stages of this disease in Canada. We have excluded from this report the 6% of cases that present with Stage IV disease, because of the multiplicity of approaches used to treat this group of patients. A subsequent report will provide estimates of the costs of treating recurrent and metastatic disease, including Stage IV disease at diagnosis, and a summation of the lifetime costs of treating breast cancer.

*Received 26 February 1998*
*Revised 30 September 1998*
*Accepted 14 October 1998*

*Correspondence to:* WK Evans

### METHODS

### Assumptions concerning the breast cancer cost analysis

A realistic evaluation of the diagnosis and management of a particular disease requires a number of simplifying assumptions. These assumptions are that: all patients in Canada have equal access to diagnosis and treatment; only diagnostic tests essential to the diagnosis of breast cancer are included and treatment patterns are representative of current ‘standard’ Canadian practice. Because the length of hospital stay is calculated to include all hospitalization up to 30 days before and 60 days after surgical intervention, the cost of important surgical complications is included. We have also estimated the frequency and cost of hospitalizations related to chemotherapy. The inclusion of hospital-related complications will capture most of the costs of treatment-related toxicities.

Our analysis includes only female breast cancers which are invasive (not in situ) and assumes that there are no major differences in the therapeutic approaches used for different tumour histologies (i.e. ductal and lobular carcinomas follow the same treatment algorithms). In addition, it is assumed that all surgical procedures (except for biopsies) are performed on an in-patient basis, to reflect standard practice in Canada in 1995. As a review of the national person-oriented database of hospital discharges (POD) indicated that breast reconstruction was performed infrequently (74 procedures for 14 000 women), it is not considered in this model, nor is the cost of care for patients entered into clinical trials (Statistics Canada, 1993–1994). Only the costs of initial management are included, which means any therapy that was initiated within the first 3 months following the diagnosis of breast cancer. Finally, the study incorporates only the direct costs associated with breast cancer management. It does not include indirect costs, such as the costs of home care, prostheses, travel and accommodation, care-givers in the home, or lost wages.

### Data sources

Many databases and registries were accessed in order to perform the breast cancer study. The Canadian Cancer Registry (CCR) at Statistics Canada was used to obtain an estimate of the incidence of Canadian women diagnosed with breast cancer in 1995. Surveys were sent to Canadian medical, surgical and radiation oncologists to determine current diagnostic, staging, therapeutic and follow-up practice patterns for Stage I, II and III breast cancer patients. The details of the methodology used to determine Canadian practice are described elsewhere (Tomiak et al, 1998). Survey results were used to augment or corroborate existing national databases.

Diagnostic, staging, treatment and survival data were obtained from several Canadian provincial breast cancer registries, including the Northern Alberta Breast Cancer Registry (1971–1988), the Saskatchewan Cancer Foundation (1985–1992), the British Columbia Cancer Agency (1989–1994), as well as a special staging study by the Manitoba Cancer Treatment and Research Foundation and the Manitoba Medical Services Foundation of all breast cancer cases diagnosed in 1990 (Sloan and Nemecek, 1995). Saskatchewan Cancer Foundation charts were retrospectively reviewed to determine current (1993) diagnostic approaches and survival, as well as the management of early stage, recurrent and metastatic disease.

For breast cancer, therapeutic options are dependent upon the stage of the disease at presentation, the age and menopausal status of the patient, and the hormone-receptor status of the tumour. Information regarding the tumour's hormone receptor status or the patient's menopausal status are not routinely available from our provincial data sources. As a result, the derived treatment algorithms are based upon the woman's age at the time of diagnosis (less than 50; or age 50 and over).

Statistics Canada's national person-oriented database of hospital discharges (POD), which includes files from April 1993 to March 1994, was used to verify the proportions of women receiving various surgical procedures, as well as the duration of hospitalization for these procedures and for terminal care (Statistics Canada, 1993–94). In order to include any hospitalization for preoperative tests and procedures, and to incorporate readmissions associated with surgical complications, all hospital admissions up to 30 days prior to, and 60 days after the admission date for surgery, were included in the length of stay calculations. Finally, many breast cancer specialists gave freely of their time to provide information, advice and expertise to verify the appropriateness of the breast cancer model in the Canadian health care environment.

### Cost assessment

All costs were determined in constant 1995 Canadian dollars and the economic analysis was carried out from the perspective of the government as payer in a universal health care system. As fee codes and amounts paid for surgical, laboratory and other procedures are different for each province in Canada, the Canadian Institute for Health Information (CIHI) was commissioned to perform a 10-province comparison of the cost of the most commonly used breast cancer tests and surgical procedures. Ultimately, the rates in the 1995 Ontario Health Insurance Plan (OHIP) were used, because they were determined to approximate closely the average cost of physician assessments, tests and surgical procedures in Canada.

In order to determine hospital resource utilization by case mix grouping (CMG), information was obtained from the Ontario Case Cost Project's (OCCP) 1993–1995 database for total mastectomy (CMG 429, 430 and 431) and partial or subtotal mastectomy (CMG 432 and 433). The OCCP uses standardized methodology to collect patient-level cost data from 13 Ontario hospitals. Weighted averages were calculated for women receiving the two main surgical options, according to age group (< 50 years; ≥ 50 years) to arrive at an appropriate per diem rate for each procedure.

The cost of chemotherapy administration included the 1995 chemotherapy and antiemetic drug acquisition costs, the cost of drug preparation and administration by pharmacy and nursing personnel (Ottawa Regional Cancer Centre), the laboratory investigations necessary to monitor patients during chemotherapy, and physicians' services and clinic overhead costs, assuming that chemotherapy was administered on an out-patient basis. Protocols from the National Surgical Adjuvant Breast and Bowel Project (NSABP) were reviewed to determine that febrile neutropenia and dehydration from vomiting were the major complications of chemotherapy administration likely to require hospitalization for treatment (NSABP Progress Report, June 1994). The amount of hospitalization that could result from these chemotherapy complications was extracted from Statistics Canada's POD. The hospital per diem rates were provided by OCCP. These calculations have been incorporated into the model.

The cost of one fraction of radiotherapy was based on a study performed at the Ottawa Regional Cancer Centre, based upon 1995/96 costs. The average cost per fraction of radiation therapy of $138 Canadian included the salaries and benefits of all staff involved in the radiation treatment programme, as well as the depreciation of radiotherapy equipment, the capital cost of the construction of the radiation treatment facilities and administrative costs (Earle et al, 1997).

Facility overhead costs for ambulatory visits to a cancer centre to receive radiotherapy or chemotherapy were extracted from an economic analysis of a National Cancer Institute of Canada clinical trial (BR-5) (Jaakkimainen et al, 1990). The cost per clinic visit for a chemotherapy or radiotherapy assessment was adjusted from $53.11 in 1984 to $76.74 in 1995, based on the increase in the CPI over that time frame. A 1996 unpublished study shows similar clinic costs for chemotherapy administration in Ontario (an average cost per visit of $65.50 compared to our cost of $76.74; Maroun, 1998). When these more recent data are published, they will be incorporated into our model.

The costs associated with tamoxifen hormonal therapy were obtained from the pharmacy departments of the Ottawa Civic and General hospitals. It was assumed that 20 mg of Tamoxifen would

© Cancer Research Campaign 1999

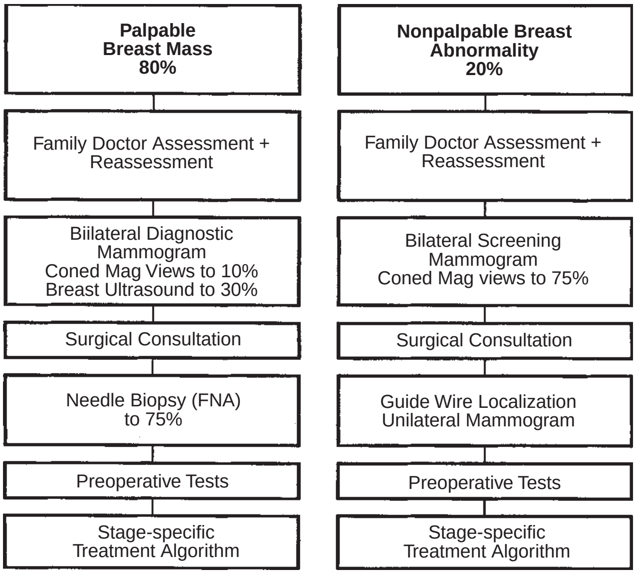

**Figure 1** Diagnostic work-up schema for breast cancer be administered daily over a period of 5 years to women requiring adjuvant hormonal therapy. For this paper on the economic burden of initial therapy, the cost of providing hormonal therapy is based on the first year costs only.

### RESULTS

According to data from the Canadian province of Saskatchewan, 46% of patients present with Stage I breast cancer and 41% have Stage II disease. Only 7% and 6% present with Stages III and IV, respectively. These data are consistent with 1992 Surveillance, Epidemiology and End Results Program (SEER) data, which indicate that 88.6% of all invasive breast cancer is diagnosed at Stages I and II, 6.8% at Stage III and 4.6% at Stage IV (Ries et al, 1997). Canadian Cancer Registry data indicate that 77% of breast cancer patients are diagnosed at age 50 or older, and that over 60% of these are between the ages of 60 and 79. Fewer than 6% of women are diagnosed with breast cancer before they reach the age of 40 (National Cancer Institute of Canada, 1997).

For ease of analysis and presentation, the management of breast cancer has been divided into modules for diagnosis, staging and treatment. There are six therapeutic algorithms: one for women less than 50 years of age at the time of diagnosis, and one for women aged 50 and over, for each of the three stages. It is assumed that each component of each treatment algorithm is self-contained and can be added to other components as the patient progresses through the course of her illness.

### Diagnostic module

The diagnostic module is based upon the results of surveys of oncologists, the Saskatchewan chart reviews, and informal input from oncologists. It includes those tests, procedures and assessments required to confirm a diagnosis of breast cancer (see Figure 1). The diagnostic module contains the assumption that a breast mass that is less than 1 cm in size (pathologically) is considered to be nonpalpable. The women who present with a nonpalpable mammographic breast abnormality (approximately 20% of cases) generally have their diagnosis established by means of a guide wire localization and biopsy. The majority (80%) of women present with a palpable breast mass and have the diagnosis of cancer confirmed with a fine needle biopsy.

Data indicate that a family physician completes the diagnostic work-up by ordering a chest X-ray, routine blood work and biochemistry tests, prior to referring the patient for a surgical consultation. Postmenopausal women also have an ECG as part of their preoperative work-up. Information taken from cancer registries and expert opinions indicates that of the 80% of women diagnosed with a palpable breast mass, all have a bilateral diagnostic mammogram, 10% of these women have coneo down magnification views and 30% have a breast ultrasound. The majority of women with a palpable mass have a fine needle biopsy. The average of the diagnostic work-up for a woman presenting with a palpable mass is $317. Nonpalpable breast abnormalities discovered by bilateral screening mammography usually require coneo down magnification views (75% of cases). It is assumed that all of these are referred for a surgical consultation and are diagnosed by means of a guide wire localization and excisional biopsy, at a total cost of $377.

### Staging assessments

All patients undergo preoperative testing and limited staging investigations. These investigations include a chest X-ray, basic blood work and biochemistry, in addition to the tests and procedures included in the diagnostic work-up. Twenty-five per cent of clinical Stage I and 75% of Stage II patients also have bone scans and abdominal ultrasounds. All Stage III patients have these staging studies. Only 5% of Stages I and II have skeletal surveys, compared with 25% of Stage III. As a result, the average cost of the staging assessment is estimated to be $142 for Stage I patients, $257 for Stage II, and $334 for Stage III.

### Therapeutic approaches

*Surgical options*

The treatment algorithms for pre- and postmenopausal patients with Stages I–III are shown in Figures 2, 3 and 4, respectively. For Stages I and II, surgery is the treatment of choice, with some variation in the rate of partial vs total mastectomy according to stage and age group. For Stage III patients, surgery is employed on 85% of women less than 50 years old and 75% of those age 50 years and older. Of the Stage III patients who undergo surgery, approximately 15% receive neoadjuvant chemotherapy.

The cost of surgery includes the surgical fee for the procedure, as well as assistant and anaesthetist fees. Pathology consultation and technical fees are not included in the cost of surgery, as they are incorporated into the calculation of hospital resource expenditure. The average cost for breast-conserving surgery (BCS) of $666 was calculated from the following distribution of procedures, based on data from Saskatchewan: 20% of women have an excisional biopsy after a mammographic wire localization; 80% undergo a partial mastectomy or a wedge resection; 5% of surgical patients have an anaesthesia consultation; specimen radiography is done routinely; and all patients undergo axillary node dissection (AND).

*© Cancer Research Campaign 1999*

The cost of a mastectomy was estimated to be $707, based on the following distribution of surgical procedures, as extracted from hospital discharge files for 1993–94: 80% of women undergoing a mastectomy have a modified radical mastectomy; 17% have a simple mastectomy; and 3% have a radical mastectomy.

### *Hospitalization*

The length of stay for breast-conserving surgery (BCS) for women less than 50 years old and women aged 50 and over, was 4.5 and 5.2, respectively, compared with 5.6 days and 6.7 days for women undergoing a mastectomy. It was assumed that one in-patient physician assessment ($17.10) would be added for each day in hospital. For those receiving a mastectomy, the average resource utilization per day for women less than 50 years of age was $741, compared with $674 for women aged 50 and over. The average daily resource utilization for all women undergoing partial mastectomies was $838. Combining the per diem rates with length of stay data, the average total cost of hospitalization for BCS was $3822 for women less than 50 and $4447 for women 50 and over. For women having a mastectomy, the costs were $4218 and $4643, respectively.

### *Radiotherapy costs*

***Following breast-conserving surgery*** As indicated in the algorithms, the majority of women who are treated with breast-conserving surgery receive postoperative radical radiotherapy. The Saskatchewan database and the survey results indicate that the usual dose is 50 Gy, which is administered at a rate of five fractions per week over a 5-week period. It is assumed that patients receive one initial consultation plus weekly partial assessments and haematological monitoring during the course of radiotherapy. Twenty per cent of Stages I and II women and 25% of Stage III receive a boost dose of 12.5 Gy in five fractions. The total cost of radiotherapy following BCS (including the boost) was estimated to be $3867 for Stages I and II and $3903 for Stage III.

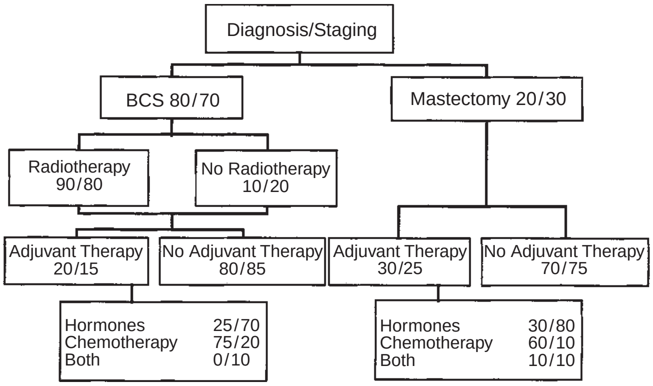

Proportions are shown for women < 50 first, and women ≥ 50 after the slash

**Figure 2** Treatment algorithm for Stage I. Per cent of women aged less than 50/ABE 50 and older (*n* = 1873/6269)

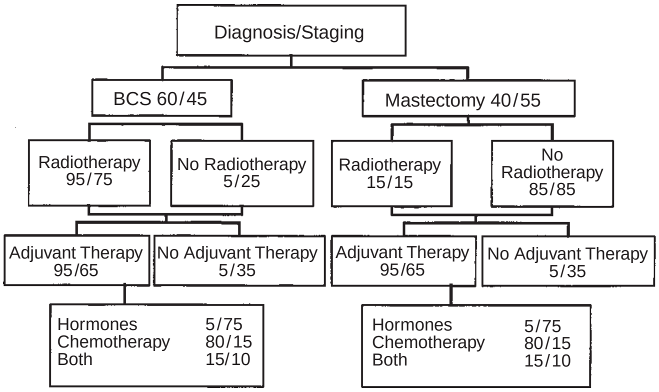

Proportions are shown for women < 50 first, and women ≥ 50 after the slash

**Figure 3** Treatment algorithm for Stage II. Per cent of women aged less than 50/ABE 50 and older (*n* = 1669 / 5588)

*© Cancer Research Campaign 1999* 

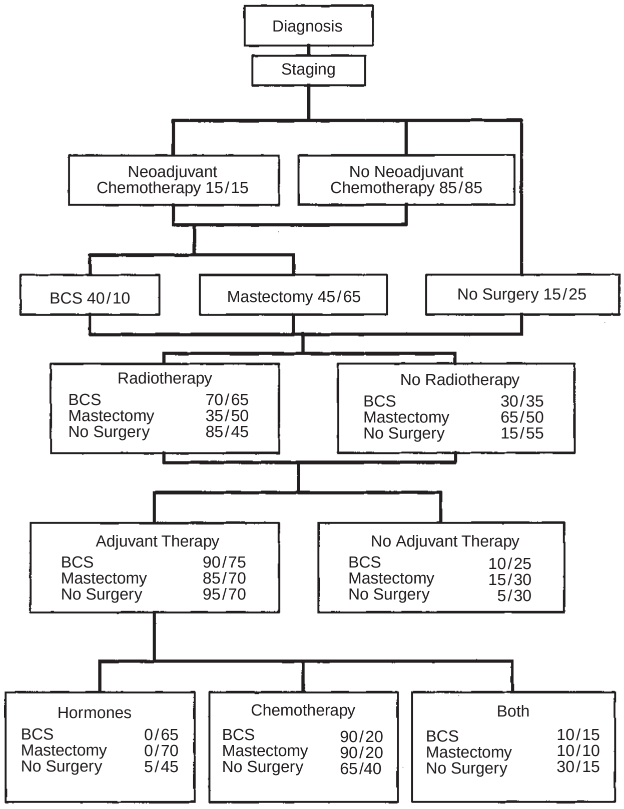

Proportions are shown for women < 50 first, and women ≥ after the slash

**Figure 4** Treatment algorithm for Stage III. Per cent of women aged less than 50/ABE 50 and older (*n* = 286/954)

***Following mastectomy*** No Stage I patients receive radiotherapy after mastectomy. However, 15% of Stage II patients receive postoperative radiotherapy and for Stage III patients, the proportion is significantly higher (35% of those < 50 years and 50% of those ≥ 50 years). The total cost of postoperative radiotherapy, based on 45 Gy administered in five fractions per week over 4 weeks, is estimated to be $2999.

### *Chemotherapy costs*

The estimates of the proportions of women receiving various adjuvant chemotherapy regimens (by stage) were based on data from the Saskatchewan chart reviews and from the survey responses of oncologists. These data show that when chemotherapy is given for early stage breast cancer (Stages I and II), the majority of patients (70 to 90%) receive six cycles of cyclophosphamide, methotrexate, and 5-fluorouracil (CMF), with a smaller proportion receiving anthracycline-based regimens [four cycles of cyclophosphamide and doxorubicin (AC) or six cycles of cyclophosphamide, doxorubicin and 5-fluorouracil (CAF)]. The cost of antiemetics has been included in the total cost of chemotherapy administration. For CMF, the usual antiemetic is 10 mg of oral prochlorperazine, whereas for anthracycline-based regimens, 8 mg each of oral ondansetron and IV dexamethasone are administered. In addition, NSABP protocols, the national database of hospital discharges, and OCCP data were used to calculate the cost of treatment-related toxicities. For 4% of those receiving CMF and 6.6% of those receiving AC or FAC, we have added 6.0 days of hospitalization at a per diem rate of $595 Canadian to take into consideration chemotherapy complications such as febrile neutropenia or dehydration due to vomiting.

In addition to physician assessment fees, blood work and biochemistry tests, it was assumed that a MUGA scan ($208) would be required for all women receiving AC and CAF. The average chemotherapy cost varied from $2376 for Stage I after BCS, to $2797 for Stage II after mastectomy. There are three chemotherapy options for Stage III patients: neoadjuvant chemotherapy, postoperative chemotherapy, and chemotherapy alone. Six cycles of CAF chemotherapy were identified as the chemotherapy regimen of choice for all options and were administered to 75% of these women at an average cost of $4088.

### *Cost of hormonal therapy*

For those patients receiving hormonal therapy, the cost of 20 mg of tamoxifen, administered daily over a period of 5 years, with an annual gynaecological assessment, biochemistry and blood work, was $427 per year. Because this paper presents only the initial costs of therapy, we show the cost of providing hormonal therapy for 1 year, instead of 5 (see Table 1).

### Summary of costs by stage and age group

The estimated average costs of diagnosis and initial treatment of Stages I, II and III breast cancer patients are shown in Table 1. It is important to note that Table 1 amalgamates the costs of the various

*© Cancer Research Campaign 1999*

**Table 1** Per patient and total costs of diagnosis and initial treatment of breast cancer (1995 Cdn $)

| | Stage I | | Stage II | | Stage III | | Weighted average |
|---|---|---|---|---|---|---|---|
| **Component** | **< 50 years** | **≥ 50 years** | **< 50 years** | **≥ 50 years** | **< 50 years** | **≥ 50 years** | **(3 stages)** |
| Diagnosis | 329 | 329 | 329 | 329 | 329 | 329 | 329 |
| Staging | 142 | 142 | 257 | 257 | 334 | 334 | 206 |
| Surgery | 674 | 678 | 682 | 688 | 589 | 530 | 671 |
| Hospitalization | 3901 | 4505 | 3982 | 4556 | 3458 | 3488 | 4325 |
| Radiotherapy | 2768 | 2177 | 2386 | 1529 | 2048 | 1640 | 2014 |
| Chemotherapy | 382 | 116 | 2419 | 446 | 4091 | 1529 | 637 |
| Hormonal therapy[a] | 29 | 68 | 86 | 243 | 47 | 222 | 133 |
| Total | 8225 | 8014 | 10 140 | 8048 | 10 897 | 8073 | 8315 |
| No. of patients[b] | 1873 | 6269 | 1669 | 5588 | 285 | 954 | 16 638 |
| Total cost ($,000) | 15 403 | 50 244 | 16 925 | 44 970 | 3105 | 7701 | 138 348 |

[a]The cost of hormonal therapy includes therapy provided in the first year only. [b]Excludes 1062 patients with Stage IV disease at presentation. Numbers may not add up due to rounding.

treatment arms. As examples, the cost of surgery is a weighted average of the cost of BCS and mastectomy; the cost of chemotherapy for Stage III patients combines the costs of neoadjuvant chemotherapy, postoperative chemotherapy, and chemotherapy alone. Table 2 provides a detailed breakdown of the costs of all the tests and procedures for a 'typical' breast cancer case. A Stage 1 postmenopausal woman receiving BCS followed by radiotherapy (but no adjuvant therapy) was chosen as an example, as these women represent 35% of all breast cancer cases. While Table 2 only shows the costs for diagnosis and initial treatment of early stage breast cancer, our comprehensive costing model for breast cancer will also contain the same level of detail for diagnostic and therapeutic options related to all other stages of breast cancer, as well as for local recurrence and metastatic disease.

The Population Health Model has been used to calculate costs according to the proportions flowing into each component of the algorithm (surgery, radiotherapy, chemotherapy, etc.), in order to arrive at an average total cost for each stage and age group. As indicated, the total average cost by stage and age group does not vary a great deal, except that it is slightly more expensive to treat younger (less than 50 years of age) women, generally because a higher proportion receive BCS followed by radiotherapy (e.g. for Stage II women < 50 years old, the total cost is $10 140, compared with $8048 for women ≥ 50 years old).

The largest cost components are hospitalization for surgery, which ranges from $3458 for Stage III women < 50 years old, to $4556 for Stage II women ≥ 50 years old; and radiotherapy, which ranges from $1529 for Stage II women ≥ 50 years old, to $2768 for

**Table 2** Component costs of a 'typical' Stage I postmenopausal breast cancer case

| **Test or procedure** | **Description and costs** | **1995 costs ($)** |
|---|---|---|
| Diagnostic module (palpable breast mass) | Family physician assessment (48.20) and reassessment (28.10), mammogram (57.34), breast ultrasound (37.80), surgical consultation (55.90), chest X-ray (30.71), CBC (10.34), biochemistry I + electrolytes (33.08), fasting blood sugar (3.10), ECG (15.50), fine needle aspiration (33.40) | 353 |
| Staging assessment | Chest X-ray (30.71), CBC (10.34), biochemistry II + electrolytes (38.25) | 79 |
| Surgical procedure (BCS) | Specimen radiography (9.84), partial mastectomy or wedge resection (336.44), radical axillary node dissection (328.12) | 674 |
| Hospitalization costs | $838/day × 5.2 days, in-hospital physician assessments (17.10 × 5.2 days) | 4447 |
| Radiotherapy after BCS | Radiation consultation (105.40), partial assessment (23.10 × 5), 25 fractions of radiotherapy (138.00 × 25), CBC (10.34 × 5) | 3653 |
| Total | | 9206 |

CBC = haemoglobin, differential + platelets. Biochemistry I = alkaline phosphatase, creatinine, SGOT (AST), LDH, bilirubin. Biochemistry II = alkaline phosphatase, creatinine, SGOT (AST), LDH, bilirubin + calcium. Electrolytes = sodium, potassium + chloride. These costs are for an individual case and cannot be compared to other costs, which are averages of various therapeutic options.

© Cancer Research Campaign 1999

Stage I women < 50 years old. Consistent with recent treatment practices, there are differences in the proportion of patients receiving systemic adjuvant chemotherapy and hormonal therapy administered, according to patient age. Chemotherapy is commonly used for Stage III women less than 50 years old and costs an average of \$4091 per patient, compared with an average cost of \$1529 for those aged ≥ 50, who tend to receive more hormonal therapy. The total estimated cost on a national level for the initial treatment of Stages I, II and III breast cancer patients is \$65.6, 61.9, and 10.8 million, respectively, differences which are attributable largely to the proportion of women falling into each of these stage groups.

### DISCUSSION

The breast cancer study has been developed from a number of comprehensive patient databases, such as the Canadian Cancer Registry and the national person-oriented database of hospital discharges. Whenever possible, these databases were compared with the results of surveys of oncologists and verified by our team of experts. A review of the reported treatment practices from the surveys of oncologists suggests that the data provided by the provincial cancer registries are reasonably representative of common Canadian treatment practice.

In cases where no national data were available, information from provincial databases was used, after it was verified. As an example, the Ontario Fee Schedule was used to determine the costs of physician assessments, and diagnostic or surgical tests and procedures. In this case, the Canadian Institute for Health Information was commissioned to perform comparative provincial analyses to ensure that these costs were representative of those at the national level.

It is recognized that there is considerable variation in practice across the country, stage for stage. For example, a study undertaken in Ontario found a wide regional variation within the province in the frequency of breast-conserving surgery (Iscoe et al, 1994). According to Iscoe and colleagues, the mean proportion of breast-conserving procedures by county was 52%, with a range from 11 to 84%. This variation was strongly associated with the hospital where the surgery was performed. Other explanations for the differences found were the woman's age at diagnosis (older women were less likely to undergo BCS) (Silliman et al, 1989; Cady and Stone, 1990; Farrow et al, 1992; Ganz, 1992; Satariano et al, 1992; Howe et al, 1995), geography (Cady and Stone, 1990; Ferguson et al, 1990; Farrow et al, 1992; Howe et al, 1995), and the availability of radiotherapy (Cady and Stone, 1990). Also, physician preference (Cady and Stone, 1990; Long, 1993), tumour size (Delouche et al, 1987; Cady and Stone, 1990; Margolese, 1995), the patient's own personal preferences (Cady and Stone, 1990), local medical customs and practices (Cady and Stone, 1990; Kiebert et al, 1991; Margolese, 1995), and co-morbidity (Satariano, 1992) all influence the choice of surgical procedure. Because we were able to use data on surgical procedures and length of stay from a national database, the treatment algorithms that we have developed incorporate the above-mentioned variations in practice patterns and surgical choices into the national average.

The authors acknowledge that diagnostic tests may be duplicated as patients move through the health care system, or if there are co-morbid conditions. While this assumption will undoubtedly lead to undercounting the economic impact of the diagnostic work-up, these costs are small, and even if doubled, would not constitute a significant component of the total expenditure on breast cancer.

Surgical treatment-related toxicities and complications during the initial treatment phase of breast cancer are taken into consideration through the extra days required for hospitalization. Our data incorporate the costs of immediate postoperative complications, as well as any readmissions up to 60 days after surgery, but do not capture out-patient follow-up or home care. This will tend to underestimate the total initial surgical treatment costs somewhat. Adjuvant chemotherapy is often associated with acute complications such as severe nausea and vomiting requiring intravenous fluid and nutritional support, or, for a small proportion of patients, febrile neutropenia, which can require hospitalization. The costs associated with these complications of treatment have been included in the study.

The surveys of oncologists were used to fill gaps where databases did not exist. We have had to assume that the oncologists' questionnaire responses were a true reflection of their practice patterns. However, it is important to bear in mind that studies have demonstrated that there is frequently a discrepancy between what physicians claim they do and what they actually do (McPhee et al, 1986; Sawka et al, 1995). In practice, treatment-related decisions are influenced by a variety of patient characteristics that are not necessarily incorporated into the scenarios presented in questionnaires.

This analysis could be criticized for including only the direct medical costs of breast cancer treatment. However, even the capture and calculation of these costs is a formidable and, at times, frustrating task, as has been observed by others (Hillner, 1993). In an attempt to compare our Canadian breast cancer costs with those of American researchers, we reviewed the work of Baker et al (1991), Taplin et al (1995) and Riley et al (1995). In all of these articles, there appears to be consensus that breast cancer costs can be separated into the cost of initial therapy, ongoing or maintenance care, and terminal care. Direct comparisons of the average cost of initial therapy for breast cancer patients are problematic as the length of the 'initial therapy' phase can vary from 3 months, as the comparator years range from 1990 to 1995 and Canadian and American currencies are used. However, it is interesting to note that whatever methodology is used, hospitalization consistently accounts for the bulk of the costs of initial therapy, and Canadian costs are generally much lower. The average cost of initial therapy in Canada in 1995 (\$8315 Cdn or \$6053 US) is considerably less than the estimates from the above-cited articles. When their costs are converted to 1995 dollars, they range from \$15 536 (Baker et al, 1991) to \$12 424 (Taplin et al, 1995) to \$11 792 (Riley et al, 1995). There are two plausible reasons for the difference in costs. It is generally acknowledged that hospital administration costs in the United States are higher than those in Canada because of different health care systems. This is attributed to the fact that hospitals require large departments to handle the claims of multiple insurers. As Canadian hospitals are part of a universal health care system, there is no such requirement. A second reason could be that the majority of hospitals in the USA are 'for profit', which is not the case in Canada.

Baker and Stommel have both attempted to calculate some of the indirect costs of cancer care. Baker et al (1991) estimated that the costs of home care and prescription drugs could increase the total cost of care by 17% in the USA. Stommel et al (1993) estimated that the average out-of-pocket costs and loss of income were similar to the costs of a nursing home bed over a 3-month period (\$4563 indirect costs vs \$5704 US dollars for a nursing home). Inclusion of Canadian costs, with appropriate sensitivity

*© Cancer Research Campaign 1999*

analyses, would give a more comprehensive measure of the full economic impact of breast cancer. However, the full burden of breast cancer cannot be determined without also taking into consideration quality of life issues, which are beyond the scope of this study.

The breast cancer study has a level of sophistication which, we believe, provides a realistic 'baseline' estimate of the cost per case of treatment by stage and therapeutic modality. This economic analysis was conducted as part of a larger effort, to provide information on various diseases for a comprehensive microsimulation model called POHEM (Population Health Model), which is under development at Statistics Canada. POHEM is designed to simulate the health status of the Canadian population, by integrating data on risk factors, disease onset and progression, health care resource utilization, direct medical care costs and health outcomes (Wolfson, 1994). POHEM currently models lung cancer and breast cancer, and contains a simple model of coronary artery disease. A colorectal cancer module is being developed, as is a more sophisticated cardiovascular disease module.

Models such as POHEM are useful tools to estimate the economic burden of common disease. For example, the POHEM lung cancer submodel developed in 1993 allowed us to evaluate the cost of the diagnosis and treatment of 'standard' Canadian practice, and then to estimate the cost and cost-effectiveness of several new therapeutic interventions (Evans et al, 1995*a*, *b*, 1996; Evans and Le Chevalier, 1996; Evans, 1996).

We are now completing a study of the therapeutic options for recurrent and metastatic breast disease. It will then be possible to estimate the lifetime costs of treating breast cancer in Canada. This will allow the breast cancer database to be used to assess the cost impact and cost-effectiveness of new diagnostic, therapeutic or preventive interventions. For example, it could be used to evaluate the cost-effectiveness of outpatient breast cancer surgery. Capri and colleagues found that the cost of the hospital stay for mastectomy in Italy was 40% greater than quadrantectomy, with an average length of stay which was longer (18 vs 14.1 days) (Capri et al, 1992). Based on 9 months of data from the 1993–94 Canadian hospital discharge database (10 580 cases), the average length of stay for mastectomy was also longer than that for BCS (6.7 days vs 5.2 for women over 50), a 30% difference. There is clearly a potential to reduce the costs of initial surgical management by reducing the number of hospital days in Canada. McManus and her colleagues analysed 118 patients, all of whom underwent either modified radical mastectomy, lumpectomy and axillary node dissection, or axillary dissection alone as outpatients. They observed only three patients who suffered minor complications requiring hospital admission. The outpatient cost for a modified radical mastectomy was $1572 US, compared with an average 3-day inpatient cost of $6282 US (McManus et al, 1994). POHEM has been used to show that the adoption of such approaches in Canada results in significant savings to the health care system (Evans et al, 1998).

In addition, the breast cancer model could be used to evaluate new diagnostic approaches such as the cost impact of 'sentinel' lymph node biopsy, as suggested by Cady (1996), or stereotactic core biopsy for nonpalpable mammographic abnormalities. Hillner suggests that stereotactic core biopsies are less invasive, avoid anaesthetic risk, cause minimal changes in breast cosmesis and have a lower initial procedure cost (Hillner, 1996).

Additionally, with the advent of newer, but significantly more expensive systemic agents (antioestrogens, cytotoxics), the impact on management costs may be assessed readily. The model is already being used to evaluate the impact of postmastectomy radiation for patients with node-positive disease, as well as the impact of hormone replacement therapy on postmenopausal women. Such information will be invaluable to guide future decisions regarding treatment policies. The development of models such as this one will make it possible to assess the interplay between risk factors, disease states and costs. It provides a valuable tool to measure the overall health status of Canadians and to analyse the impact of health policies on the Canadian population.

### ACKNOWLEDGEMENTS

The long process of developing a disease model requires cooperation, input and feedback from numerous individuals and organizations. We wish to thank Dr Michael Wolfson for his contribution in the development of POHEM, and acknowledge the dedication of our colleagues Christian Houle and Bill Flanagan, in the Health Analysis and Modelling Group, who analyse and synthesize the raw data for incorporation into the POHEM microsimulation model. We are also indebted to the staff of the Health Statistics Division of Statistics Canada, who maintain our national cancer registry and the national person-oriented database of hospital discharges, and who provide invaluable advice.

We could not have completed this project without the cooperation of several provincial cancer registries, which provided us with much-needed data and feedback. We have made extensive use of the data provided through a contractual arrangement with the Saskatchewan Cancer Foundation and owe Diane Robson and her staff a special acknowledgement. In addition, we were fortunate to obtain valuable information from the Northern Alberta Breast Cancer Registry, and wish to thank Dr Alan Lees and Heather Jenkins. Through Dr Paul Blood and Jeff Sloan, we obtained the data from a 1990 staging study of breast cancer done by the Manitoba Cancer Treatment and Research Foundation and the Manitoba Medical Services Foundation. Thanks to Dr Ivo Olivotto and his staff, we received staging data from the British Columbia Cancer Registry. In addition, we must acknowledge the time and effort of the surgical, radiation and medical oncologists across Canada, who responded (without financial remuneration) to a lengthy questionnaire prepared under the auspices of the Ottawa Regional Cancer Centre.

We wish to acknowledge the comparative analysis of provincial fee schedules done for us by Jill Strachan at the Canadian Institute for Health Information, the costing and length of stay chart abstractions performed by Ken Potvin and Lisa Gregoire at the Ottawa General Hospital, and the data provided by the Ontario Case Cost Project. Personnel at the Ottawa Regional Cancer Centre are to be thanked for providing us with chemotherapy drug and supply costs, in addition to the pharmacy and nursing personnel time.

Special thanks go to Dr Diane Logan, who provided much-needed information, advice and feedback during the initial stages of this project; Dr Doug Mirsky, who dedicated a large amount of time to walk us through the intricacies of breast cancer surgical procedures; Dr Peter Cross, who helped us in finalizing the radiation therapy aspects of the model; and to Dr Paul Blood, who attempted to educate some of the nonmedical members of the team and guided our efforts with his sage advice.

Finally, we owe a special debt to Rolande Belanger, Christine Allen, Monica Bongers and Sharon Tessier for their administrative support throughout this project.

*© Cancer Research Campaign 1999* 

### REFERENCES

Baker MS, Kessler LG, Urban N and Smucker RC (1991) Estimating the treatment costs of breast and lung cancer. *Med Care* **29**: 40–49

Cady B and Stone MD (1990) Selection of breast-preservation therapy for primary invasive breast carcinoma. *Surg Clin North Am* **70**: 1047–1059

Cady B (1996) Cost-effective preoperative evaluation, operative treatment, and postoperative follow-up in the breast cancer patient. *Surg Clin North Am* **76**: 25–34

Capri S, Majno E and Mauri M (1992) The cost of hospital stay for operable breast cancer. *Tumori* **78**: 359–362

Delouche G, Bachelot F, Premont M and Kurtz JM (1987) Conservation treatment of early breast cancer: long term results and complications. *Int J Radiat Oncol Biol Phys* **13**: 29–34

Earle C, Coyle D, Smith A, Agboola O and Evans WK (1997) The cost of radiotherapy at an Ontario regional cancer centre. *Eur J Cancer* **33**(Suppl 9): S4 (abstr. OP9)

Evans WK, Will BP, Berthelot J-M and Wolfson MC (1995*a*) Diagnostic and therapeutic approaches to lung cancer in Canada and their costs. *Br J Cancer* **72**: 1270–1277

Evans WK, Will BP, Berthelot J-M and Wolfson MC (1995*b*) Estimating the cost of lung cancer diagnosis and treatment in Canada: the POHEM model. *Can J Oncol* **5**: 408–419

Evans WK, Will BP, Berthelot J-M and Wolfson MC (1996) The economics of lung cancer management in Canada. *Lung Cancer* **14**: 19–29

Evans WK and Le Chevalier T (1996) The cost-effectiveness of Navelbine alone or in combination with cisplatin in comparison to other chemotherapy regimens and best supportive care in stage IV non-small cell lung cancer. *Eur J Cancer* **32A**: 2249–2255

Evans WK (1996) An estimate of the cost-effectiveness of Gemcitabine in stage IV non-small cell lung cancer. *Semin Oncol* **23**(Suppl 10): 82–89

Evans WK, Le Petit C, Will BP, Berthelot J-M, Tomiak EM, Verma S and Logan D (1998) Strategies to reduce the costs of care for breast cancer in Canada. Oral presentation, International Society for Technology Assessment in Health Care, Ottawa, June 1998

Farrow DC, Hunt WC and Samet JM (1992) Geographic variation in the treatment of localized breast cancer. *N Engl J Med* **326**: 1097–1101

Ferguson CM, Feinstein AC and Pendergrast WJ (1990) Determinants of primary therapy of early stage breast cancer. *J Med Assoc Ga* **79**: 351–354

Ganz PA (1992) Treatment options for breast cancer — beyond survival. *N Engl J Med* **326**: 1147–1149

Hillner BE (1993) Financial costs, benefits, and patient risk preferences in node-negative breast cancer: insights from a decision analysis model. *Recent Results Cancer Res* **127**: 277–284

Hillner BE (1996) Economic and cost-effectiveness issues in breast cancer treatment. *Semin Oncol* **23** (Suppl 2): 98–104

Howe HL, Johnson TP, Lehnherr M, Warnecke RB, Katterhagen JG and Ford L (1995) Patterns of breast cancer treatment: a comparison of a rural population with an urban population and a community clinical oncology program sample. *Cancer Control* 113–120 (March/April)

Iscoe NA, Goel V, Wu K, Fehringer G, Holowaty EJ and Naylor CD (1994) Variation in breast cancer surgery in Ontario. *Can Med Assoc J* **150**: 345–352

Jaakkimainen L, Goodwin PJ, Pater J, Warde PM, Murray N and Rapp E (1990) Counting the costs of chemotherapy in a National Cancer Institute of Canada randomized trial in non-small cell lung cancer. *J Clin Oncol* **8**: 1301–1309

Kiebert GM, de Haes JC and van de Velde CJ (1991) The impact of breast-conserving treatment and mastectomy on the quality of life of early-stage breast cancer patients: a review. *J Clin Oncol* **9**: 1059–1070

Long DS (1993) How breast cancer patients choose a treatment method. *Radiol Technol* **65**: 30–33

Margolese RG (1995) 1994 Roussel Lecture: a biological basis for changes in breast cancer management. *Can J Surg* **38**: 402–408

Maroun J (1998) The cost of chemotherapy delivery. Oral presentation, Department of Oncology, Queen's University, Canada, February 1998

McManus SA, Topp DA and Hopkins C (1994) Advantages of outpatient breast surgery. *Am Surg* **60**: 967–970

McPhee S, Richard R and Solkowitz S (1986) Performance of cancer screening in a university general internal medicine practice: comparison with the American Cancer Society guidelines. *J Gen Intern Med* **1**: 275–281

National Cancer Institute of Canada (1995) *Canadian Cancer Statistics 1995*. Toronto, Canada

National Cancer Institute of Canada (1997) *Canadian Cancer Statistics 1997*. Toronto, Canada

National Cancer Institute of Canada (1998) Canadian Cancer Statistics 1998. Toronto, Canada

Ries LAG, Kosary CL, Hankey BF, Miller BA, Harras A and Edwards BK, eds (1997) *SEER Cancer Statistics Review*: *1973–1994, National Cancer Institute*. NIH. Pub No. 97-2789, Bethesda, MD

Riley GF, Potosky AL, Lubitz JD and Kessler LG (1995) Medicare payments from diagnosis to death for elderly patients by stage at diagnosis. *Med Care* **33**: 828–841

Satariano ER, Swanson GM and Moll PP (1992) Nonclinical factors associated with surgery received for treatment of early-stage breast cancer. *Am J Public Health* **82**: 195–198

Satariano WA (1992) Comorbidity and functional status in older women with breast cancer: implications for screening, treatment, and prognosis. *J Gerontol* **47**: 24–31

Sawka CA, O'Connor AM, Llewellyn-Thomas HA, To T, Pinfold SP and Harrison-Woermke D (1995) Appropriateness of adjuvant systemic therapy for axillary node-negative breast cancer: a physician opinion survey. *J Clin Oncol* **13**: 1459–1469

Silliman RA, Guadagnoli E and Weitberg AB (1989) Age as a predictor of diagnostic and initial treatment intensity in newly diagnosed breast cancer patients. *J Gerontol* **44**: M46–50

Sloan J and Nemecek A (1995) Experiences in constructing cancer patient trajectories through the Manitoba health care system. Final report to the Manitoba Medical Services Foundation, July 1995

Statistics Canada (1993–94) *National Person-oriented Database of Hospital Discharges, 1993–1994*

Statistics Canada (1995) *Annual Demographic Statistics, 1995*. Catalogue no. 91-213-XPD: 188

Stommel M, Given CW and Given BA (1993) The cost of cancer home care to families. *Cancer* **71**: 1867–1874

Tabar L, Fagerberg G, Day NE, Duffy SW and Kitchin RM (1992) Breast cancer treatment and natural history: new insights from results of screening. *Lancet* **339**: 412–414

Taplin SH, Barlow W, Urban N, Mandelson MT, Timlin DJ, Ichikawa L and Nefcy P (1995) Stage, age, comorbidity, and direct costs of colon, prostate, and breast cancer care. *J Natl Cancer Inst* **87**: 417–426

Tomiak EM, Diverty B, Verma S, Evans WK, Le Petit C, Will BP and Berthelot J-M (1998) Follow-up practices for patients with early stage breast cancer: a survey of Canadian oncologists. *Cancer Prevention & Control* **2**: 63–71

Will BP, Berthelot J-M, Houle C, Verma S, Tomiak E and Evans WK (1993) A model for estimating the costs and burdens of breast cancer diagnosis and treatment in Canada. *Health Rep* **5**: 399–408

Wodinsky H and Jenkin RD (1987) The cost of radiation treatment at an Ontario regional cancer centre. *Can Med Assoc J* **137**: 906–909

Wolfson MC (1994) POHEM – a framework for understanding and modelling the health of human populations. *World Health Statist Quart* **47**: 157–176

*© Cancer Research Campaign 1999*
